# Solid-State Fermentation of *Arthrospira platensis* to Implement New Food Products: Evaluation of Stabilization Treatments and Bacterial Growth on the Volatile Fraction

**DOI:** 10.3390/foods10010067

**Published:** 2020-12-30

**Authors:** Francesco Martelli, Martina Cirlini, Camilla Lazzi, Erasmo Neviani, Valentina Bernini

**Affiliations:** Department of Food and Drug, University of Parma, Parco Area delle Scienze 49/A, 43124 Parma, Italy; francesco.martelli@studenti.unipr.it (F.M.); camilla.lazzi@unipr.it (C.L.); erasmo.neviani@unipr.it (E.N.); valentina.bernini@unipr.it (V.B.)

**Keywords:** *Arthrospira platensis*, fermentation, lactic acid bacteria, food supplement, aromatic profile

## Abstract

*Arthrospira platensis* is a cyanobacterium widely used in food formulation and mainly consumed as a food supplement because of its high amount of proteins, vitamins and minerals. Different probiotic food supplements are present in the market, and a lactic acid fermented food product like dried spirulina could be useful not only to introduce lactic acid bacteria (LAB) with beneficial effects to the diet of consumers, but also to improve or change the aromatic profile of the substrate. Therefore, the aim of this study was the evaluation of lactic acid fermentation of *A. platensis* biomass, focusing on the consequent changes in the aromatic profile. For this purpose, two different stabilization treatments (UV light treatment and sterilization) were applied prior to fermentation with two LAB strains, *Lacticaseibacillus casei* 2240 and *Lacticaseibacillus rhamnosus* GG. The biomass proved to be a suitable matrix for solid-state fermentation, showing a LAB growth of more than 2 log CFU/g in 48 h. The fermentation process was also useful for off-flavor reduction. In particular, the fermentation process significantly influenced the concentration of those compounds responsible for aldehydic/ethereal, buttery/waxy (acetoin and diacetyl), alkane and fermented aromatic notes (isoamyl alcohol). The heat treatment of the matrix, in addition to guaranteed safety for consumers, led to an improved aroma after fermentation. In conclusion, a fermented spirulina powder with a different aromatic profile was obtained with the applied heat treatment. Fermentation with lactic acid bacteria can be an interesting tool to obtain cyanobacterial biomasses with more pleasant sensory properties for potential use in food formulations.

## 1. Introduction

*Arthrospira platensis*, commercially known as Spirulina, is a cyanobacterium commonly consumed as a food supplement because of its high nutritional value [1,2]. It is characterized by a high percentage of proteins (60%), providing all of the essential amino acids, followed in abundance by carbohydrates and polyunsaturated fatty acids (ω-3 and ω-6). Moreover, discrete quantities and varieties of minerals, vitamins and pigments (C-phycocyanin, chlorophyll and carotenoids) [3] are present. To date, microalgae and spirulina, defined as the novel food of the future [4], are used for various purposes in nutraceutical, cosmetic [5], feed [6] and pharma [7] sectors, and they continue to increasingly attract the interest of consumers and companies because of several bioactivities that this cyanobacterium is being proved to possess in many studies [8,9]. Applications with the aims of conferring macro- and micro-nutrients and improving color are increasing, and many food products, such as bread, cookies and pasta [10,11,12], cheeses [13], yogurt [14,15] and beverages [16], supplemented with *A. platensis* have been developed. To date, most of the algal biomass produced is consumed in the form of powder or tablets as protein- and micronutrient-rich supplements. Positive results have also been obtained using this cyanobacterium as a prebiotic for lactic acid bacteria (LAB). Indeed, it was able not only to preserve LAB viability in food matrixes [17,18] but also to stimulate their growth in broth [19,20,21]. The effect of *A. platensis* biomass on LAB could be exploited for the production of probiotic food supplements or ingredients in particular. Fortification of foods with probiotic strains shows beneficial effects such as anti-inflammatory, antioxidant and immunomodulatory effects; protection against colitis; and damage to epithelial cells [22]. Moreover, probiotics, once ingested, are believed to play an important role in the intestinal tract against foodborne pathogens [23] and can reduce symptoms due to antibiotic therapies, thereby relieving food allergies and reducing atypical dermatitis [24]. A lactic acid fermentation process applied to spirulina dried powder could be useful not only to supply consumers of LAB with beneficial effects to their diet, but also to improve or change the aromatic profile as seen in the case of vegetable matrices [25]. Indeed, a biological process to reduce unpleasant smells may represent an important tool for applications of algal biomass in complex foods for the avoidance of off-flavors. During fermentation, the metabolic activity of LAB leads not only to rapid acidification of the substrate and fast consumption of easily fermentable sugars, with a competitive advantage in the use of LAB in nutrient-rich environments, but also to the production of volatile compounds belonging to different chemical classes, such as alcohols, aldehydes, ketones, acids, esters and sulfur compounds. These compounds mainly derive from the catabolism of citrate and from the degradation of proteins and lipids. The formation of aromatic compounds is a complex process in which precursors are initially generated and subsequently converted into aromatic compounds [26].

On the other hand, complex issues arise in the production of spirulina-based products that can be considered safe for consumer health. Because of cultivation conditions and manipulation during downstream processing, *A. platensis* can be contaminated by alterative and pathogenic bacteria [27] that may duplicate during fermentation, thus compromising quality and safety.

The aromatic fraction of *A. platensis* is characterized by several volatiles present in different concentrations and with different odor thresholds formed in the matrix during growth and maturation [28]. The most abundant volatiles identified as aromatic components of *A. platensis* are hydrocarbons, especially heptadecane, followed by furanic compounds, pyrazines, sulfur compounds, aldehydes, ketones and alcohols [28,29,30]. All of these substances, naturally present in the matrix and ascribed to amino acid and fatty acid microorganism metabolism, contribute to the typical fishy odor that characterizes algae and algae-derived products [31,32]. This unpleasant flavor could be reduced by applying a fermentation step to the product, as described in recent works in which yeasts and bacteria were used to ferment different algae matrixes [30,33].

On the basis of these considerations, the aim of the present study was to evaluate solid-state fermentation of *A. platensis* biomass considering two LAB species, *Lacticaseibacillus rhamnosus* and *Lacticaseibacillus casei*, belonging to the *Lacticaseibacillus casei* group, for the production of a lactic acid-fermented food product. In order to reach this goal, to stabilize the biomass, two different treatments based on UV light irradiation and thermal sterilization were applied prior to fermentation. The effects on LAB growth were evaluated and the aromatic fraction of *A. platensis* was characterized in order to assess the improvements in the volatile profile given by LAB fermentation.

## 2. Materials and Methods

### 2.1. Arthrospira Platensis Stabilization Treatments

The dehydrated *A. platensis*, kindly provided by Bertolini Farm (Fidenza, PR, Italy), was used as a substrate for fermentation. It is a commercial product cultivated in raceway pond and marketed as “Organic Spirulina” according to EU Organic Aquaculture Regulation (EC No. 834/07). In order to reduce the microbial contamination present on the biomass, two different treatments were applied separately. The first consisted of UV light irradiation for 15 min to reduce the microbial total charge applied under a Faster BH-EN 2004 Class II Microbiological Safety Cabinet (S/N 1113) (Richmond Scientific, Chorley, UK) with a lamp emitting light at an intensity of 253.7 nm in the spectrum of UV-C (UV), while the second was based on a thermal treatment of 121 °C for 20 min applied in an autoclave (3870MLV, Tuttnauer, NY, USA) to sterilize the algal biomass (ST). Specifically, the time for the complete sterilization cycle was of approximately 50 min. To reach the temperature of 121 °C, 15 min were necessary, while 20 min were required to perform the sterilization process and a further 15 min to cool the autoclave after treatment. To evaluate the efficacy of the stabilization treatments, microbial plate counts were performed following treatments and after 48 h of incubation at 37 °C in order to determine the residual microbial load. To this purpose, samples were ten-fold serially diluted in Ringer solution (Oxoid, Basingstoke, UK) and plated on plate count agar (PCA) (Oxoid, Basingstoke, UK) incubated at 37 °C for 48 h. Analyses were performed in duplicate and average values ± standard deviations were reported as log CFU/g.

### 2.2. Lactic Acid Bacteria Strains

Two LAB strains were used to ferment *A. platensis* biomass: *Lacticaseibacillus casei* 2240, isolated from Parmigiano Reggiano cheese, belonging to the collection of the Department of Food and Drug (University of Parma), and *Lacticaseibacillus rhamnosus* GG, a probiotic commercial strain. They were maintained at −80 °C in de Man, Rogosa and Sharpe (MRS) cultivation medium (Oxoid, Basingstoke, UK), with 12.5% glycerol (*v/v*) added before use.

### 2.3. Arthrospira Platensis Biomass Fermentation

LAB strains were revitalized twice in MRS broth (Oxoid) (inoculation of 3% *v/v*) incubated for 16 h at 37 °C under aerobic conditions. They were then inoculated in fresh MRS broth (3% *v/v*) and incubated for 15 h at 37 °C to obtain a bacterial concentration of 9 log CFU/mL. After centrifugation (Eppendorf centrifuge 5810 R, Eppendorf, Hamburg, Germany) (12,857× *g* for 10 min at 4 °C), cells were collected, washed twice in Ringer solution (Oxoid, Milan, Italy) and suspended in sterile bidistilled water.

*A. platensis* biomass was rehydrated with 70% *w/w* sterile water, and then inoculated individually with each bacterial suspension in order to obtain an estimated LAB concentration of 7 log CFU/mL in each sample. LAB concentration was evaluated following the inoculation (T0), and after 24 h (T1) and 48 h of fermentation (T2). Serial dilutions of the samples in Ringer (Oxoid) were plated on MRS agar (Oxoid) and incubated for 48 h at 37 °C in aerobic conditions. pH of all the samples, before and after the fermentation step, was also measured (Mettler Toledo, Greifensee, Switzerland). Fermentations were carried out in duplicate, and for each sampling time analyses were performed in duplicate. Colonies were counted manually, and to calculate the CFU/g concentration, the following equation was applied:

(1) $$\frac{\Sigma c}{\left(1\times na+0.1\times nb+0.01\times nc\right)d}$$

where *∑C* is the summation of all the counted colonies, *na* is the number of plates of the first countable serial dilution, *nb* is the number of plates of the second countable serial dilution, *nc* is the number of plates of the third countable serial dilution, and *d* is the serial dilution factor of the first countable serial dilution.

Average values ± standard deviation were reported as log CFU/g. Treated but not inoculated samples were also incubated at 37 °C and analyzed at the same sampling times as control samples. The 48 h fermented and stabilized but not fermented samples were then lyophilized by a Freeze dryer Lio-5P (5Pascal, Milan, Italy) for 48 h, and then the LAB concentration was determined by plate counting on MRS.

### 2.4. HS-SPME/GC-MS Analysis

The characterization of the volatile fraction was conducted on all the fermented and stabilized but not fermented *A. platensis* samples. To this purpose, the protocol reported by Ricci et al. 2019 [25] was applied with some modifications. Briefly, 2 g of biomass and 5 µL of an aqueous Toluene standard solution (100 μg/mL in 10 mL) were used for the analyses. The headspace of the samples was extracted by a divinylbenzene–Carboxen–polydimethylsiloxane (DVB/Carboxen/PDMS) SPME fiber (Supelco, Bellefonte, PA, USA) for 30 min at 40 °C after an equilibration time of 15 min at the same temperature. The separation of the volatile compounds was achieved using a SUPELCOWAX 10 capillary column (Supelco, Bellefonte, PA, USA; 30 m × 0.25 mm × 0.25 μm) placed on a Thermo Scientific Trace 1300 gas chromatograph coupled with a Thermo Scientific ISQ single quadrupole mass spectrometer equipped with an electronic impact (EI) source. All the parameters applied for the analyte separation and detection, such as the injector, transfer line and column compartment temperatures, injection mode and gas carrier flow, were the same as those reported by Ricci et al. 2019 [25]. The detected volatile compounds were then identified on the basis of their linear retention indexes, calculated using as reference a C8–C20 alkane solution analyzed under the same chromatographic conditions applied for sample analysis, and by the comparison of the registered mass spectra with those reported in the instrument libraries (NIST 14). The semiquantification of all the identified volatiles was achieved on the basis of the use of a reference compound (Toluene). Data were reported as µg/g of wet weight.

### 2.5. Statistical Analysis

In order to determine the actual growth of tested LAB strains in *A. platensis* samples, results obtained from microbial counts were statistically treated applying one-way ANOVA test, comparing different growing times and lyophilized samples.

One-way ANOVA was carried out also considering data obtained from semiquantification of all the detected volatiles to underline analogies and differences among the considered samples in terms of production/diminution and/or release of volatiles. Moreover, two-way ANOVA was applied in order to evaluate the influence of two different factors, fermentation and stabilization treatment, on the volatile profile. All the analyses were performed using IBM SPSS Statistics 23.0 software (SPSS Inc., Chicago, IL, USA) applying Tukey’s test as a post hoc test (*p* ≤ 0.05).

## 3. Results and Discussion

### 3.1. Arthrospira Platensis Fermentation

One of the aims of the study was the evaluation of solid-state fermentation of *A. platensis* with *L. casei* bacteria that could lead not only to the implementation of new lactic acid-fermented food products but also to modifications of algae biomass aromatic fraction. Fermenting a matrix having a high bacterial charge could negatively affect the finished product considering that spoilage and/or pathogenic bacteria may also grow during the process. In this specific case, the biomass utilized for the experiment was cultivated in a raceway pond. This type of cultivation is associated with undesired microflora, such as *Bacillus* spp., *Alteromonas* spp., *Flavobacterium* spp. and *Pseudomonas* spp. [27], and previous studies have reported a variable concentration of bacteria, ranging from 2 to 7 log CFU/g, in this type of matrix [3]. For this reason, prior to fermentation, carried out for 48 h in sterilized glass cans, samples were subjected to UV radiation treatment (which does not seem to affect *A. platensis* composition [34]) or sterilization treatment in an autoclave in order to reduce the presence of microbiological contaminants. The UV treatment was ineffective for the reduction of microbial contamination, which was maintained at the same level of untreated samples (5.10 ± 0.2 log CFU/g). In order to predict the UV disinfection rates on food surfaces, it is necessary to consider the interactions between microorganisms and surface materials while trying to avoid the shielding effects from incident UV and predict the dependency on the surface structure or topography [35]. For this reason, UV treatment did not reduce the initial microbial contamination because of an uneven distribution of rays in the dehydrated samples. Furthermore, the presence of a high amount of pigments, phenolic compounds and other components with a protective effect against UV rays may contribute to microbial survival [36]. After UV treatment, a fermentation process of 48 h was conducted considering two *Lacticaseibacillus casei* bacteria. *L. casei* 2240 and *L. rhamnosus* GG concentrations were determined following inoculation, and growth was determined after 24 and 48 h of incubation at 37 °C. The endogenous contamination did not affect the counts, giving a much more higher LAB inoculation of 7 log CFU/g.

**Figure 1:**
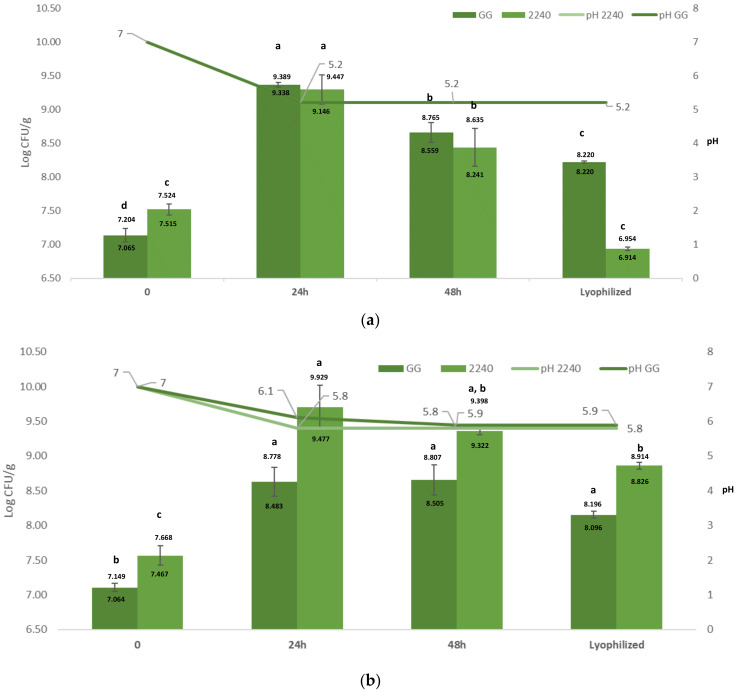
Lactic acid bacteria (LAB) growth (log CFU/g) and pH values in *Arthrospira platensis* biomass after 24 and 48 h of fermentation at 37 °C for *Lacticaseibacillus rhamnosus* GG (GG) and *Lacticaseibacillus casei* 2240 (2240) bacteria: (**a**) UV-treated samples; (**b**) heat-treated samples. Data are represented as average ± SD (bars); two replicates for each sample were measured. Letters indicate significant differences (*p* < 0.05).

As shown in (Figure 1A), the two strains showed a similar growth trend in the UV-treated matrix. After 24 h of fermentation, both species showed good replication capacity, reaching a concentration higher than 9 log CFU/g. In particular, *L. rhamnosus* GG increased by 2.23 log CFU/g (from 7.13 ± 0.09 to 9.36 ± 0.03 log CFU/g; *p* < 0.05), and to a higher extent than *L. casei* 2240 with a growth of 1.78 log CFU/g (from 7.52 ± 0.06 to 9.30 ± 0.21 log CFU/g).

After 48 h of fermentation, a decrease in the concentration of both strains was observed. *L. casei* 2240 decreased by 0.86 log CFU/g (from 9.30 ± 0.21 log CFU/g to 8.44 ± 0.28 log CFU/g), while *L. rhamnosus* GG decreased by 0.70 log CFU/g (from 9.36 ± 0.03 log CFU/g to 8.66 ± 0.14 log CFU/g) (Figure 1A) (*p* < 0.05). However, the values remained higher than the inoculation. The *L. casei* 2240 strain has previously been used to ferment other matrixes, such as *Himanthalia elongata*, but the growth ability on this brown seaweed was lower than that observed in this study [37]. The best capacity of this strain to grow on *A. platensis* can be linked to the high amount of proteins and small peptides [2]. *L. rhamnosus* GG is a probiotic commonly supplemented to fermented milk [38], but applications to other matrices have also been evaluated. For instance, a sausage supplemented with *L. rhamnosus* GG has been produced [39] though this strain grew to a lesser extent than in *A. platensis*.

During fermentation, the initial pH of the hydrated *A. platensis* biomass decreased from 7.0 ± 0.1 to 5.2 ± 0.1 in the first 24 h for both samples and remained stable until the end of the fermentation process. This decrease in pH during fermentation reflects the growth of the LAB strains with consequent production of organic acids, mainly lactic acid.

Considering the possibility to use lactic acid-fermented *A. platensis* in a food supplement formulation or as an ingredient, the survival of LAB was evaluated after a lyophilization process.

*L. rhamnosus* GG was reduced at 8.22 ± 0.02 log CFU/g (*p* < 0.05), and *L. casei* 2240 was reduced by more than 1 log CFU/g (from 8.44 ± 0.28 to 6.9 ± 0.03 log CFU/g) (*p* < 0.05) (Figure 1A).

The second fermentation process was carried out on *A. platensis* samples treated at 121 °C for 20 min in order to eliminate the presence of any microbial contamination. The absence of residual contamination after treatment was confirmed by plate count below the detection limit (1 log CFU/g). The sterilization treatment involves the use of a particular apparatus, the autoclave, and high energy to reach the temperatures and pressures required by the process. This treatment is therefore longer and more expensive than that based on UV rays, but the results show that it is certainly more effective in reducing the initial microbial concentration of the product. Following the heat treatment, the characteristic green-blue color of *A. platensis* changed to a dark green, brownish color. This may be due to the degradation of pigments such as carotenoids, xanthophylls, phycocyanins and chlorophyll caused by the high temperatures reached [40,41,42]. The growth trend of LAB strains is presented in (Figure 1B). In particular, after 24 h, *L. casei* 2240 increased by 2.13 log CFU/g (from 7.57 ± 0.142 to 9.70 ± 0.320 log CFU/g) (*p* < 0.05) and *L. rhamnosus* GG by 1.5 log (from 7.10 ± 0.06 to 8.63 ± 0.21 log CFU/g) (*p* < 0.05) and then remained constant (with no significant differences) up to 48 h.

The ability of these *Lacticaseibacillus* species to grow on different matrixes has been challenged over the years, with different results [25,39,43,44]. In particular *L. casei* and *L. rhamnosus* strains grown on sterilized vegetables’ by-products increased by more than that observed on *A. platensis* [45]. However, the ability of this cyanobacterium to boost the growth of LAB is known in the literature [20], and because of its richness in small peptides and proteins, *A. platensis* can be considered a good matrix to allow for the growth of LAB species such as *L. casei* and *L. rhamnosus*.

After fermentation of the sterilized biomass, a lower acidification rate than that of the UV-treated fermented samples was observed. The pH value of the sterilized fermented biomasses after 24 h was 5.8 ± 0.1 and 6.1 ± 0.1, respectively, for *L. casei* 2240 and *L. rhamnosus* GG and also remained stable at 48 h of fermentation (Figure 1B).

Analogously to the UV-treated samples, a lyophilization process was applied to the sterilized products after fermentation. A decrease in the microbial concentration of 0.5 log CFU/g was observed for both strains; in particular, *L. rhamnosus* GG was reduced to 8.15 ± 0.05 log CFU/g and *L. casei* 2240 to 8.86 ± 0.05 log CFU/g (*p* < 0.05) (Figure 1B).

*L. rhamnosus* GG better survived the lyophilization process than *L. casei* 2240, whose viability showed a decrease of more than 1 log CFU/g. The survivability of freeze-dried strains is of particular importance for the production of foods containing live cells, and, for this reason, many studies focus on enhancing the viability of LAB after this process [46,47]. The better survivability of *L. casei* 2240 to lyophilization in sterilized biomass than UV-treated samples was observed on the basis of the applied statistical model (Figure 1). Several authors [17,48,49] tested the addition of *A. platensis* to dairy products, such as yogurt, cheese and fermented milk, with positive results, including an increase in the number of LAB and an improvement in the nutritional quality of the fermented product during storage.

**Figure 2:**
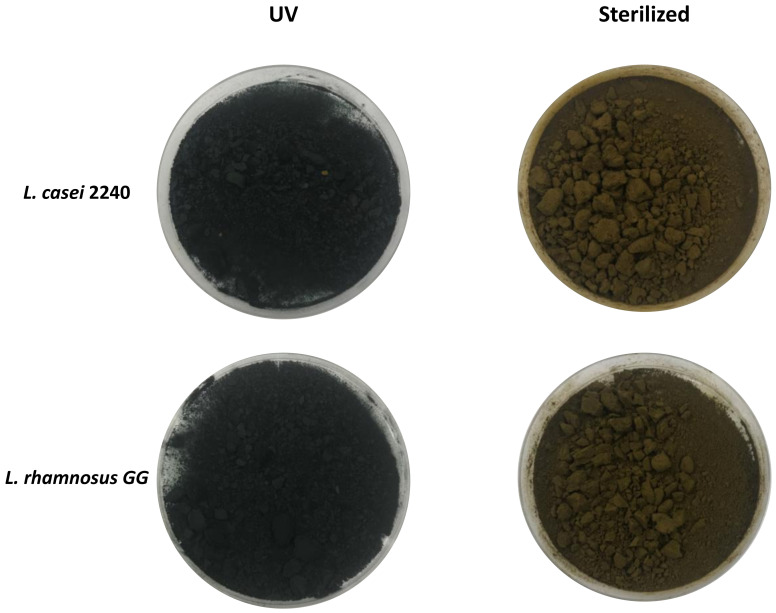
Representative image of fermented and lyophilized *Arthrospira platensis* biomasses subjected to UV and sterilization treatments.

The fermentation process leads to increased production of phenolic compounds and phycocyanobilins and consequently increased radical scavenger properties of the cyanobacterium [50]. Lactic acid fermentation is an appropriate method to enhance the functional properties of spirulina and also to integrate beneficial bacteria into consumers’ diets, thereby providing further advantages to the final product. However, to meet the definition of probiotic products, microorganisms must be viable for the entire shelf life of the product, and in such quantities to be able to multiply and integrate the intestinal flora. The activity of *A. platensis* to enhance LAB vitality, such as that of *L. casei, Streptococcus thermophilus, Lacticaseibacillus acidophilus*, and *Bifidobacteria,* has been documented [3,19,20,21]. There is no consensus regarding the minimum quantity of probiotic microorganisms to be ingested to guarantee their functionality in the human intestine. Usually, to observe a positive effect on health, 6 to 7 log CFU/g of live probiotic microorganisms, able to colonize the intestine, should be consumed daily [51]. All of the fermented biomasses obtained in this study presented a sufficient amount of bacteria, allowing the production of fermented foods with a high functional value. An image of the fermented biomasses produced after lyophilization is presented in Figure 2.

### 3.2. Volatile Profile Characterization of A. platensis and Changes in Volatile Components after Fermentation

Recently, several studies have been conducted on the characterization of the aromatic fraction of *A. platensis*, and, in particular, a number of studies were carried out with the aim of identifying how the characteristic fishy odor of this product can be reduced in order to use it as additive or ingredient in food [29,30,33]. The reduction of unpleasant aromatic notes can be achieved by solvent extraction [29], such as by fermentation using fungi or bacteria [30,33]. The deodorization of *A. platensis* can be performed by applying an extraction procedure to the biomass; Cuellar-Bermúdez et al. stated that the fishy odor can be reduced by treating spirulina with a solvent (e.g., ethanol, acetone or hexane) capable of dissolve aromatic compounds, thereby preserving the nutritional characteristic of the product [29]. On the other hand, fermenting *A. platensis* with lactic acid bacteria, such as *Lacticaseibacillus plantarum* or *Bacillus subtilis,* may remove the volatile compounds initially present in the matrix and lead to the formation of new components, such as acetoin, that provide fermented and creamy aromatic notes [30]. In the present study, lactic acid bacteria fermentation was applied to stabilized *A. platensis* materials, and changes in the volatile fraction were determined.

A total of 61 different volatile compounds were identified in the volatile fraction of treated but not fermented and fermented *A. platensis* samples (Table 1). In particular, 7 aldehydes, 9 ketones, 4 esters, 9 terpenes/norisoprenoids, 7 alcohols, 4 furans, 11 hydrocarbons, 7 pyrazines and 3 sulfur compounds were detected. These results are consistent with data reported by Bao et al. (2018), who detected the same classes of volatile compounds in spirulina samples fermented with different strains of *L. plantarum*, *L. acidophilus* and *Bacillus subtilis* [30].

The class that quantitatively mainly represents the aromatic fraction of *A. platensis,* both before and after the fermentation process, is that of hydrocarbons (Supplementary Table S1), as also demonstrated in previous studies [30]. The concentration of these compounds was higher in respect to the amount of all the other components. Hydrocarbon release was significantly different when comparing fermentations (*p* = 0.007) and opposing technological treatments (*p* < 0.001).

**Figure 3:**
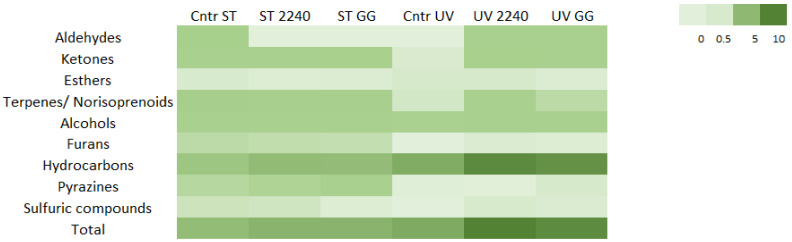
Heat map performed on volatile chemical classes’ concentrations detected in sterilized (ST) or UV-treated (UV) *A. platensis* biomasses fermented with *L. casei* 2240 (2240) and *L. rhamnosus* GG (GG) as well as treated but unfermented biomasses (Cntr). A scale ranging from a maximum of 10 µg/g (dark green) to a minimum of 0 µg/g (light green) was used.

Statistical differences were observed in hydrocarbon quantity among the UV- and heat-treated samples (*p* < 0.001), among samples subjected to the same process, and between fermented and not fermented samples (*p* = 0.007), but no interaction between factors (type of treatment and fermentation) was observed (Figure 3, Supplementary Table S1).

**Table 1:** Volatile compounds found on *Arthrospira platensis* treated but not fermented and fermented samples. For each volatile compound’s aromatic note, calculated and tabulated linear retention indices (LRIs), references and effect given by treatments, stabilization and fermentation (statistical difference = positive = p; no statistical difference = negative = n; not determinable = nd) are reported.

Chemical Class, Compound Name	Odor Type	Calculated LRI	Reference LRI	Identification Method	Reference	Effect of Stabilization	Effect of Fermentation
*Aldehydes*							
Isobutyraldehyde	aldehydic	805	814	MS + LRI	[52]	n	n
2-Methylbutanal	chocolate	904	903	MS + LRI	[53]	nd	nd
Isovaleraldehyde	aldehydic	907	888	MS + LRI	[54]	p	p
Hexanal	green	1075	1086	MS + LRI	[55]	n	n
Methional	vegetable	1452	1468	MS + LRI	[56]	p	p
Benzaldehyde	fruity	1523	1537	MS + LRI	[55]	n	n
2,5-Dimethyl benzaldehyde		1733	1705	MS + LRI	[53]	p	p
*Ketones*							
Acetone	solvent	810	901	MS + LRI	[52]	p	p
2-Butanone	ethereal	894	901	MS + LRI	[52]	p	p
diacetyl	buttery	971	973	MS + LRI	[54]	n	p
6-Methyl-2-heptanone	camphoreous	1229	1236	MS + LRI	[57]	p	p
3-Octanone	herbal	1245	1261	MS + LRI	[58]	n	p
2-Octanone	earthy	1277	1287	MS + LRI	[53]	p	n
Acetoin	buttery	1282	1300	MS + LRI	[54]	n	p
2,2,6-Trimethylcyclohexanone	thujonic	1306	1308	MS + LRI	[59]	p	n
Sulcatone	citrus	1329	1335	MS + LRI	[60]	p	n
*Esters*							
Ethyl acetate	ethereal	872	869	MS + LRI	[61]	n	p
Ethyl caprylate	waxy	1430	1438	MS + LRI	[61]	nd	nd
Ethyl decanoate	waxy	1628	1645	MS + LRI	[61]	p	n
Phenethyl acetate	floral	1804	1803	MS + LRI	[62]	nd	nd
*Terpenes, norisoprenoids and similar*							
p-Xylene		1133	1149	MS + LRI	[63]	p	n
Myrcene	spicy	1155	1143	MS + LRI	[54]	n	n
α-Cyclocitral	citrus	1427	1420	MS + LRI	[53]	p	p
β-Cyclocitral	tropical	1609	1612	MS + LRI	[59]	p	n
Safranal	herbal	1635	1637	MS + LRI	[59]	p	p
α-Ionene	fruity	1675		MS		n	n
α-Ionone	floral	1841	1848	MS + LRI	[64]	p	p
β-Ionone	floral	1918	1935	MS + LRI	[64]	n	n
β-Ionone-5,6-epoxide	fruity	1950	1989	MS + LRI	[53]	p	n
*Alcohols*							
Ethanol	alcoholic	923	903	MS + LRI	[54]	p	p
Isobutyl alcohol	ethereal	1080	1100	MS + LRI	[60]	n	n
Isoamyl alcohol	fermented	1195	1210	MS + LRI	[60]	p	p
1-Pentanol	fermented	1239	1260	MS + LRI	[55]	p	n
1-Hexanol	herbal	1341	1357	MS + LRI	[61]	p	n
1-Octen-3-ol	earthy	1437	1455	MS + LRI	[55]	p	n
Benzyl alcohol	floral	1882	1896	MS + LRI	[61]	n	n
*Furans*							
2-Methylfuran	chocolate	853	876	MS + LRI	[52]	p	p
3-Methylfuran		881	877	MS + LRI	[53]	n	n
2-Butylfuran	spicy	1123	1140	MS + LRI	[65]	p	p
2-Pentylfuran	fruity	1220	1232	MS + LRI	[64]	p	n
*Hydrocarbons*							
1,2,4,4-Tetramethylcyclopentene		920		MS		n	n
2,2,4,6,6-Pentamethylheptane		944		MS		n	n
Ethyl benzene		1119	1127	MS + LRI	[66]	n	n
Tridecane	alkane	1300	1300	MS + LRI	[56]	p	p
Tetradecane	waxy/alkane	1396	1400	MS + LRI	[56]	p	p
2,6,10-Trimethyltridecane		1434	1442	MS + LRI	[53]	n	n
Pentadecane	waxy	1492	1500	MS + LRI	[56]	p	p
Hexadecane	alkane	1590	1600	MS + LRI	[56]	p	p
N-acetyl-4(H)-Pyridine		1644		MS		p	p
Heptadecane	alkane	1687	1700	MS + LRI	[56]	p	p
6,9-Heptadecadiene		1743				p	p
*Pyrazines*							
2-Methylpyrazine	nutty	1267	1267	MS + LRI	[52]	n	n
2,5-Dimethylpyrazine	chocolate	1318	1321	MS + LRI	[52]	p	n
2-Methyl-5-ethylpyrazine	coffee	1368	1406	MS + LRI	[52]	n	n
2-Ethyl-6-methylpyrazine	potato	1383	1402	MS + LRI	[52]	p	p
Trimethyl pyrazine	nutty	1398	1401	MS + LRI	[52]	p	p
2,3-Dimethyl-5-ethylpyrazine	burnt	1452	1460	MS + LRI	[53]	p	n
Tetramethyl pyrazine	nutty	1468	1474	MS + LRI	[52]	p	n
*Sulfur compounds*							
Dimethyl disulfide	sulfurous	1063	1073	MS + LRI	[54]	p	n
2-Ethyl-4-methylthiazole	nutty	1336	1322	MS + LRI	[52]	n	n
Dimethyl trisulfide	alliaceous	1369	1375	MS + LRI	[54]	n	n

Pentadecane, hexadecane and heptadecane are hydrocarbons derived from the decarboxylation of palmitic and stearic acids, respectively [29,67], and they were the most representative among all of the hydrocarbons identified in this work. In particular, heptadecane was the volatile found in the highest amount in all of the considered samples as observed in a recent study focused on the fermentation of spirulina using *Lacticaseibacillus plantarum* and *Bacillus subtilis* [30]. The presence of this compound could contribute to the off-flavor of algae associated with crude fish notes, despite the fact that it presents a high odor threshold [29]. Heptadecane was significantly lower in the sterilized samples when compared to the UV-treated ones, despite the fact that the fermentation process seemed to increase its concentration in both cases; indeed, both considered factors, stabilization treatment (*p* < 0.001) and fermentation (*p* = 0.006), appeared to influence its release/formation. In other studies considering *L. plantarum* and *B. subtilis,* the fermentation caused a 27% reduction in the relative content of heptadecane, but no information regarding the actual quantity found in the samples was reported [30]. The LAB species considered in the present work, *L. casei* and *L. rhamnosus*, provided significantly different results in the production of heptadecane in the differently treated substrates, which is indeed linkable to LAB fermentation [68]. It can be speculated that the production and/or release of this compound may depend on the applied LAB species. The same phenomenon can be observed also for pentadecane. A higher amount was detected in the UV-treated samples when compared to the sterilized ones. It can be observed that the quantity of hydrocarbon grew following fermentation when both the strains were used (Supplementary Table S1). The significant difference observed in the fermented biomasses (i.e., heptadecane: UV 2240 = 7.65 ± 1.95 µg/g; ST 2240 = 2.53 ± 0.66 µg/g) can be ascribed to the different stabilization processes applied; the higher concentration of these volatiles in the UV-treated biomass could be ascribed to the metabolism of the endogenous microflora typical of the spirulina biomass. Since the UV treatment, unlike the heat treatment, did not completely eliminate contaminating microflora, this behavior can be ascribed to the residual microflora deriving from cultivation in open ponds.

Terpenes, the second most important class in terms of concentration, were positively influenced by sterilization treatment, and an interaction between the two factors was observed (*p* = 0.013). Terpenes and norisoprenoids are fundamental compounds in food aroma. β-cyclocitral and β-ionone, with pleasant fruity and floral notes, are two volatiles typical of cyanobacteria. The formation of these compounds is due to the oxidation of carotenoids following carotene oxygenases. Norcarotenoids are an important group of compounds formed by several species of cyanobacteria, generated by enzymatic degradation of carotenes and carotenoids. For example, the oxygenase reaction of carotenoids was first described in Microcystis. β-cyclocitral is formed by the cleavage reaction of β-carotene catalyzed by the enzyme [59]. β-ionone and norcarotenoids of the β-ionone-type are an important group of compounds that were found in axenic cyanobacterial cultures and a monoxenic culture of *Phormidium* sp. [69]. Significant differences in the presence of β-cyclocitral, the compound that mainly characterizes the class of terpenes and norisoprenoids, were found because of the biomass stabilization treatment (*p* < 0.001), while no significant differences were found in β-ionone production.

Regarding the production of aldehydes and ketones and/or release, both fermentations and treatments produced significant differences in the samples (*p* < 0.02); in addition, a strong interaction was noticed between the two factors for these two volatile classes (*p* < 0.005). While differenced were noted among the two stabilization treatments (*p* = 0.012), ketone concentration was mainly affected by fermentation, (Figure 3, Supplementary Table S1). In particular for aldehydes, a reduction following the fermentation of the sterilized biomass using both the tested strains was observed. Conversely, by fermentation of the UV-treated biomass, the amount of aldehydes increased. This behavior could be due to the presence of the epiphytic bacteria typical of the spirulina biomass. Considering these data, it can be suggested that fermentative LAB can reduce the amount of these compounds, but the typical microflora of *A. platensis* can produce aldehydes, thereby giving the fermented biomass a different aroma profile. Hexanal was found in several microalgae [70,71,72,73]. Fermentation of sterilized biomass by both of the strains led to a significant diminution of hexanal content (*p* < 0.01), while only fermentation of the *L. rhamnosus* GG strain seemed to produce this compound in UV-treated samples. Since the presence of C6 aldehydes may be associated with fish odor [32], this can be considered an undesired aromatic notes, especially in certain food preparations, such as those of dairy products; thus, fermentation could provide a valuable improvement of in aromatic profile of the sample. Methional was found in both of the UV-treated fermented samples, suggesting, as already suggested by other studies [74,75], that this aldehyde is produced by LAB. This compound was also previously isolated from *Rhodomonas*, another microalgae [71]. Both fermentation and treatments applied prior to it, as well as their combination, produced significant differences in methional production (*p* < 0.001). Benzaldehyde is a typical aroma formed in several species of microalgae by enzymatic and chemical degradation of phenylalanine (generating benzaldehyde) [71]. This compound is often associated with pleasant notes of almond. It was found in the sterilized *A. platensis* biomass, and it was produced by the fermentation process applied to the UV-stabilized samples without differences among the two strains used. A similar trend was noticed for isovaleraldehyde (Supplementary Table S1), an aldehyde produced by LAB via the metabolization of the amino acid leucine [76].

Regarding ketones, a growing amount of these compounds was noticed following the fermentation of both the tested biomasses. In general, the number of ketones in sterilized biomass was higher than in the UV-treated biomass, proving the role of thermal treatment in the formation of these compounds (Supplementary Table S1). Diacetyl and acetoin are two typical aromatic compounds produced during fermentation with the characteristic buttery aroma [77]. Fermentation produced significant differences in the presence of these molecules. In particular, the fermentation with *L. casei* 2240 produced a higher amount of diacetyl compared to *L. rhamnosus* GG (*p* < 0.05) in both the UV-treated and sterilized materials. Production of these ketones derives from metabolization of pyruvate and citrate and is dependent on the strains used for fermentation [78]. Sulcatone is a citrus-like aroma. This compound was found after sterilization of the biomass with a significant difference when compared to the UV-treated samples, and its concentration was maintained after the fermentation step with both the strains. Interestingly, the presence of this aroma was never linked to *A. platensis* biomass or microalgae in general.

Fermentation was the only factor affecting the total content of alcohols and esters. Alcohols were found in all of the analyzed samples. The most representative was 1-octen-3-ol with the typical mushroom aroma, previously isolated from the microalgae aromatic fraction [72]. The statistical model underlined that this compound was significantly improved after sterilization when compared to UV-treated but not fermented samples (0.13 ± 0.03 and 0.04 ± 0.00 µg/g, respectively), but fermentation did not affect its amount. Significant differences were found for ethanol. A significantly higher amount of ethanol was found in the UV-treated biomasses compared to the heat-treated samples (*p* = 0.001). The higher amount of this alcohol in the samples could be linked to the sterilization treatment that affected the composition of the starting biomass and therefore the presence of precursors for the production of alcohols, or to the presence of epiphytic bacteria and yeasts that survived the UV treatment. Significant differences were also found in the production of ethanol during fermentation (*p* = 0.03). Both *L. casei* and *L. rhamnosus* are heterofermentative species and produced slight amounts of ethanol in the biomass during fermentation. Isoamyl alcohol, a volatile compound typical of beverages and fermented foods, is formed from leucine during fermentation [72]. This alcohol was found in sterilized samples following lactic acid fermentation. Although isoamyl alcohol has also been reported in phototrophic cultures of *Chlorella vulgaris* [71], in this study, it was not found in treated biomass, but rather in unfermented *A. platensis* biomass.

The UV treatment and the sterilization process produced significant differences in the total amount of furans, pyrazines and sulfur compounds.

Four furans were found as a component of the aromatic profile of fermented *A. platensis* biomass. 2-Pentylfuran is an important product of lipid degradation and is responsible for beany and licorice-like sensory qualities in various food products [79]; it has been associated with the typical beany, green and metallic odor of spirulina, and it was detected in considerable percentages of about 10% in fermented spirulina samples in a previous study [30]. In our case, this furan was formed following the sterilization of the biomass (0.10 ± 0.04 µg/g), but it decreased following fermentation, while in UV-treated samples, it was not detected in the not fermented substrate, but it increased after fermentation.

Pyrazines are molecules typical of roasted and thermally treated foods. Seven pyrazines were found in the analyzed samples, and most of them were connectable to the sterilization process. For example, 2,5-dimethylpyrazine, a pyrazine with a typical chocolate aroma, was found in the sterilized biomass and not in the UV-treated samples. Interestingly, some of these compounds were also produced following lactic acid fermentation. Small amounts of 2-methylpyrazine were produced by *L. rhamnosus* GG (0.02 ± 0.00 µg/g) in the sterilized biomass. This is not the first time that the production of pyrazine by *L. casei* group bacteria was underlined [80]. Among all of the pyrazines detected in the tested samples, 2,5-dimethylpyrazine and 2-methylpyrazine are of particular interest, because these compounds have been associated with the off-flavor of *A. platensis* [30].

Similar behavior was observed for sulfur compounds, prevalently found in the sterilized samples. Dimethyl disulfide is a sulfuric compound formed following thermal oxidation of other volatile sulfur compounds such as methanethiol [72]. The number of sulfur compounds seemed to be related especially to the stabilization treatment, as the concentration of these compounds was higher in the sterilized samples when compared to those treated with UV (*p* = 0.019). Generally, sulfur compounds may contribute to the unpleasant odor of algae products because of their low threshold value. Seo et al. (2017) stated that sulfides were responsible for about 26% of the total odor profile of sea tangle extract, and fungal fermentation lead to a total reduction of dimethyl disulfide after two days from the inoculation [33]. In our case, the fermentation step did not reduce the amount of this compound detected only in UV-treated materials (Supplementary Table S1).

Esters, with floral and waxy notes, are the category that presented the lower quantities in the analyzed samples. Four compounds were identified, and this is in line with what has been reported in other studies [30,33]. The stabilization treatment did not seem to affect their content, while fermentation led to a decrease in the initial quantity (*p* = 0.021) (Supplementary Table S1).

### 3.3. Sensory Properties of the Detected Volatile Components

**Figure 4:**
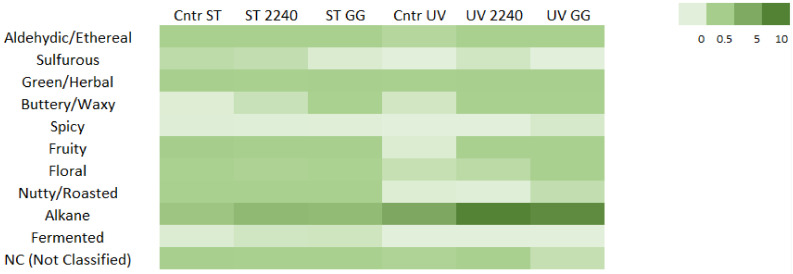
Heat map performed on the volatile concentration of volatiles grouped on the basis of their odor type detected in sterilized (ST) or UV-treated (UV) *A. platensis* biomasses fermented with *L. casei* 2240 (2240) and *L. rhamnosus* GG (GG) as well as treated but unfermented biomasses (Cntr). A scale ranging from a maximum of 10 µg/g (dark green) to a minimum of 0 µg/g (light green) was used.

In order to evaluate the modifications in the sensory properties of fermented *A. platensis* in respect to the unfermented materials and to determine the changes in aromatic notes due to the different stabilization processes applied, all of the volatile compounds and their relative concentrations were grouped based on the odor type that they were able to provide. The following ten main different aroma attributes were identified: aldehydic/ethereal, sulfurous, green/herbal, buttery/waxy, spicy, fruity, floral, nutty/roasted, alkane and fermented. On the basis of this classification, analogies and differences in odor type production among different samples were observed by applying a two way ANOVA test considering fermentation and stabilization treatments as factors (Figure 4, Supplementary Table S2). Lactic acid fermentation of spirulina biomass caused an enhancement in the aromatic profile.

In particular, the fermentation process significantly influenced the concentration of those compounds responsible for aldehydic/ethereal, buttery/waxy, alkane and of course fermented aromatic notes (*p* < 0.01). As expected, buttery/waxy and fermented notes increased with the fermentation process (Figure 4). These aromatic properties can be mainly associated with the presence of diacetyl, acetoin (buttery/waxy) and isoamyl alcohol (fermented). The concentration of all these volatiles showed an improvement in both of the stabilized materials after the fermentation process, but while in sterilized spirulina, *L. casei* 2240 produced a higher amount of diacetyl and acetoin, in UV-treated material, *L. rhamnosus* GG affected the content of these compounds more prominently (Supplementary Table S1). In addition, buttery/waxy and fermented aromatic notes were also influenced by stabilization treatment; in particular, a statistically significant interaction was observed for fermented notes of the two considered factors (*p* < 0.001) (Supplementary Table S2). Moreover, aldehydic/ethereal and alkane attributes, provided mainly by the presence of aldehydes and hydrocarbons, were strongly influenced by the fermentation step, especially in UV-treated samples (Figure 4). It must be emphasized that hydrocarbons, and in particular heptadecane, could be associate with the off-flavor of algae [41]. Alkane attribute concentrations were also influenced by the applied stabilization method (*p* < 0.01), as clearly represented in Figure 4. Thus, to limit the formation of these sensory attributes, sterilization must be chosen as the stabilization treatment.

On the other hand, significant differences were found in the production of sulfurous, green/herbal, fruity, and nutty/roasted notes (*p* < 0.02) among the stabilization treatments (Supplementary Table S2). The concentrations of all of these aromatic classes were statistically higher in the sterilized samples (*p* < 0.02). High temperature can cause the formation of pyrazine and sulfur compounds associated with nutty/roasted and sulfurous notes, as well as the degradation of carotenoids and fatty acids leading to an increase in hexanal, methional, 1-hexanol and 1-octen-3-ol (green/herbal and earthy notes), and benzaldehyde, β-cyclocitral, β-ionone 5,6-epoxide and other compounds associated with fruity aromatic notes. In addition, a statistically significant interaction between these two factors was noted for green/herbal and fruity notes (*p* < 0.001). Since fruity attributes are generally considered as pleasant aromatic properties, in this case, it is also possible to speculate that sterilization may represent the better stabilization treatment because it leads to an augment of this odor class [59,69,77,78].

In conclusion, despite the fact that sterilization may lead to some modifications of the aromatic characteristic of the starting material, *A. platensis*, it seemed to be the best choice to reduce the initial microbial count naturally occurring in the tested samples. Fermentation by *L. casei* bacteria can generate or enhance some volatile compounds responsible for pleasant aromatic notes, such as fruity and creamy (buttery) notes, associated with volatiles produced by LAB metabolism.

## 4. Conclusions

In this study, *A. platensis* biomass, treated by UV or sterilization at 121 °C, was fermented by LAB (*L. casei 2240* and *L. rhamnosus GG*) in order to evaluate the fermentative and aromatic potential. The LAB growth was not affected by the applied treatment, confirming *A. platensis* as a fermentable substrate that may be used for the development of new fermented foods and supplements with high functional values. Furthermore, the survivability of LAB to freeze-drying may allow the production of food products which, in addition to integrating proteins and vitamins typical of *A. platensis*, also include LAB or probiotic bacteria in the diet.

Considering the importance of aroma and flavor for consumers’ acceptability, the main volatile compounds involved in *A. platensis* biomass fermentation were screened. An overall improvement of smell was obtained.

In particular, a greater presence of fermentation aroma was found during the fermentation of the sterilized sample, highlighting that in addition to guaranteeing safety for the consumer, this process may enhance applications, thereby avoiding side effects due to off-flavor. In conclusion, a fermented lyophilized spirulina powder with an interesting aromatic profile and high LAB concentration was obtained with the applied heat treatment, opening up perspectives for new applications in food productions.

## Supplementary Materials

The following are available online at https://www.mdpi.com/2304-8158/10/1/67/s1, Table S1: Concentration (µg/g) of compounds identified in UV-treated and sterilized *Arthrospira platensis* fermented with *L. casei* 2240 and *L. rhamnosus* GG and in controls (not fermented UV-treated and sterilized *Arthrospira platensis*) after 48 h. Table S2: Concentration (µg/g) of odor type identified in UV-treated and sterilized *Arthrospira platensis* fermented with *L. casei* 2240 and *L. rhamnosus* GG, and in controls (not fermented UV-treated and sterilized *Arthrospira platensis*) after 48 h.

## References

[1] Batista A.P., Niccolai A., Fradinho P., Fragoso S., Bursic I., Rodolfi L., Biondi N., Tredici M.R., Sousa I., Raymundo A. (2017). Microalgae biomass as an alternative ingredient in cookies: Sensory, physical and chemical properties, antioxidant activity and in vitro digestibility. Algal Res..

[2] Andrade L.M., Andrade C.J., Dias M., Nascimento C.A., Mendes M.A. (2018). *Chlorella* and *Spirulina* Microalgae as Sources of Functional Foods, Nutraceuticals, and Food Supplements; an Overview. MOJ Food Process. Technol..

[3] Niccolai A., Shannon E., Abu-Ghannam N., Biondi N., Rodolfi L., Tredici M.R. (2019). Lactic acid fermentation of *Arthrospira platensis* (spirulina) biomass for probiotic-based products. J. Appl. Phycol..

[4] Zarbà C., Chinnici G., D’Amico M. (2020). Novel Food: The Impact of Innovation on the Paths of the Traditional Food Chain. Sustainability.

[5] Ariede M.B., Candido T.M., Jacome A.L.M., Velasco M.V.R., de Carvalho J.C.M., Baby A.R. (2017). Cosmetic attributes of algae—A review. Algal Res..

[6] Holman B.W.B., Malau-Aduli A.E.O. (2013). *Spirulina* as a livestock supplement and animal feed: *Spirulina* supplementation in livestock. J. Anim. Physiol. Anim. Nutr..

[7] Hamed I., Özogul F., Özogul Y., Regenstein J.M. (2015). Marine Bioactive Compounds and Their Health Benefits: A Review *Compr*. Rev. Food Sci. Food Saf..

[8] Martelli F., Cirlini M., Lazzi C., Neviani E., Bernini V. (2020). Edible Seaweeds and Spirulina Extracts for Food Application: In Vitro and In Situ Evaluation of Antimicrobial Activity towards Foodborne Pathogenic Bacteria. Foods.

[9] Bancalari E., Martelli F., Bernini V., Neviani E., Gatti M. (2020). Bacteriostatic or bactericidal? Impedometric measurements to test the antimicrobial activity of *Arthrospira platensis* extract. Food Control.

[10] Massoud R., Khosravi-Darani K., Nakhsaz F., Varga L. (2017). Evaluation of physicochemical, microbiological and sensory properties of croissants fortified with *Arthrospira platensis* (Spirulina). Czech J. Food Sci..

[11] Ak B., Avşaroğlu E., Işık O., Özyurt G., Kafkas E., Uslu L. (2016). Nutritional and Physicochemical Characteristics of Bread Enriched with Microalgae *Spirulina platensis*. Int. J. Eng. Res. Appl..

[12] Zouari N., Abid M., Fakhfakh N., Ayadi M.A., Zorgui L., Ayadi M., Attia H. (2011). Blue-green algae (*Arthrospira platensis*) as an ingredient in pasta: Free radical scavenging activity, sensory and cooking characteristics evaluation. Int. J. Food Sci. Nutr..

[13] Golmakani M.-T., Soleimanian-Zad S., Alavi N., Nazari E., Eskandari M.H. (2019). Effect of Spirulina (*Arthrospira platensis*) powder on probiotic bacteriologically acidified feta-type cheese. J. Appl. Phycol..

[14] Mohammadi-Gouraji E., Soleimanian-Zad S., Ghiaci M. (2019). Phycocyanin-enriched yogurt and its antibacterial and physicochemical properties during 21 days of storage. LWT.

[15] Yamaguchi S.K.F., Moreira J.B., Costa J.A.V., de Souza C.K., Bertoli S.L., Carvalho L.F.D. (2019). Evaluation of Adding *Spirulina* to Freeze-Dried Yogurts Before Fermentation and After Freeze-Drying. Ind. Biotechnol..

[16] Camacho F., Macedo A., Malcata F. (2019). Potential Industrial Applications and Commercialization of Microalgae in the Functional Food and Feed Industries: A Short Review. Mar. Drugs.

[17] Guldas M., Irkin R. (2010). Influence of *Spirulina platensis* powder on the microflora of yoghurt and acidophilus milk. Mljekarstvo.

[18] Barkallah M., Dammak M., Louati I., Hentati F., Hadrich B., Mechichi T., Ayadi M.A., Fendri I., Attia H., Abdelkafi S. (2017). Effect of *Spirulina platensis* fortification on physicochemical, textural, antioxidant and sensory properties of yogurt during fermentation and storage. LWT.

[19] Bhowmik D., Dubey J., Mehra S. (2009). Probiotic Efficiency of *Spirulina platensis*-Stimulating Growth of Lactic Acid Bacteria. World J. Dairy Food Sci..

[20] Martelli F., Alinovi M., Bernini V., Gatti M., Bancalari E. (2020). *Arthrospira platensis* as Natural Fermentation Booster for Milk and Soy Fermented Beverages. Foods.

[21] Parada J. (1998). Lactic acid bacteria growth promoters from *Spirulina platensis*. Int. J. Food Microbiol..

[22] Bron P.A., Kleerebezem M., Brummer R.-J., Cani P.D., Mercenier A., MacDonald T.T., Garcia-Ródenas C.L., Wells J.M. (2017). Can probiotics modulate human disease by impacting intestinal barrier function?. Br. J. Nutr..

[23] Gardiner G.E., Bouchier P., O’Sullivan E., Kelly J., Kevin Collins J., Fitzgerald G., Paul Ross R., Stanton C. (2002). A spray-dried culture for probiotic Cheddar cheese manufacture. Int. Dairy J..

[24] Wong S.-S., Quan Toh Z., Dunne E.M., Mulholland E.K., Tang M.L., Robins-Browne R.M., Licciardi P.V., Satzke C. (2013). Inhibition of *Streptococcus pneumoniae* adherence to human epithelial cells in vitro by the probiotic *Lacticaseibacillus rhamnosus* GG. BMC Res. Notes.

[25] Ricci A., Cirlini M., Maoloni A., Del Rio D., Calani L., Bernini V., Galaverna G., Neviani E., Lazzi C. (2019). Use of Dairy and Plant-Derived Lactobacilli as Starters for Cherry Juice Fermentation. Nutrients.

[26] Smid E.J., Kleerebezem M. (2014). Production of Aroma Compounds in Lactic Fermentations. Annu. Rev. Food Sci. Technol..

[27] Wang H., Zhang W., Chen L., Wang J., Liu T. (2013). The contamination and control of biological pollutants in mass cultivation of microalgae. Bioresour. Technol..

[28] Aguero J., Lora J., Estrada K., Concepcion F., Nunez A., Rodriguez A., Pino J.A. (2003). Volatile Components of a Commercial Sample of the Blue-Green Algae *Spirulina platensis*. J. Essent. Oil Res..

[29] Cuellar-Bermúdez S.P., Barba-Davila B., Serna-Saldivar S.O., Parra-Saldivar R., Rodriguez-Rodriguez J., Morales-Davila S., Goiris K., Muylaert K., Chuck-Hernández C. (2017). Deodorization of *Arthrospira platensis* biomass for further scale-up food applications. J. Sci. Food Agric..

[30] Bao J., Zhang X., Zheng J.-H., Ren D.-F., Lu J. (2018). Mixed fermentation of *Spirulina platensis* with *Lacticaseibacillus plantarum* and *Bacillus subtilis* by random-centroid optimization. Food Chem..

[31] Högnadóttir Á. (2000). Flavor Perception and Volatile Compounds in Fish. http://www.matis.is/media/matis/utgafa/Skyrsla_01-00_Flavor_Perception_and_Volatile_Compounds_in_Fish.pdf.

[32] Fink P. (2007). Ecological functions of volatile organic compounds in aquatic systems. Mar. Freshw. Behav. Physiol..

[33] Seo Y.-S., Bae H.-N., Eom S.-H., Lim K.-S., Yun I.-H., Chung Y.-H., Jeon J.-M., Kim H.-W., Lee M.-S., Lee Y.-B. (2012). Removal of off-flavors from sea tangle (*Laminaria japonica*) extract by fermentation with *Aspergillus oryzae*. Bioresour. Technol..

[34] Pala Ç.U., Toklucu A.K. (2011). Effect of UV-C light on anthocyanin content and other quality parameters of pomegranate juice. J. Food Compos. Anal..

[35] Koutchma T. (2008). UV Light for Processing Foods. Ozone Sci. Eng..

[36] De Jesus Raposo M., de Morais A., de Morais R. (2016). Emergent Sources of Prebiotics: Seaweeds and Microalgae. Mar. Drugs.

[37] Martelli F., Favari C., Mena P., Guazzetti S., Ricci A., Rio D.D., Lazzi C., Neviani E., Bernini V. (2020). Antimicrobial and Fermentation Potential of *Himanthalia elongata* in Food Applications. Microorganisms.

[38] Jia R., Chen H., Chen H., Ding W. (2016). Effects of fermentation with *Lacticaseibacillus rhamnosus* GG on product quality and fatty acids of goat milk yogurt. J. Dairy Sci..

[39] Rubio R., Aymerich T., Bover-Cid S., Guàrdia M.D., Arnau J., Garriga M. (2013). Probiotic strains *Lacticaseibacillus plantarum* 299V and *Lacticaseibacillus rhamnosus* GG as starter cultures for fermented sausages. LWT Food Sci. Technol..

[40] Antelo F.S., Costa J.A.V., Kalil S.J. (2008). Thermal degradation kinetics of the phycocyanin from *Spirulina platensis*. Biochem. Eng. J..

[41] Dutta D., Dutta A., Raychaudhuri U., Chakraborty R. (2006). Rheological characteristics and thermal degradation kinetics of beta-carotene in pumpkin puree. J. Food Eng..

[42] Weemaes C.A., Ooms V., Van Loey A.M., Hendrickx M.E. (1999). Kinetics of Chlorophyll Degradation and Color Loss in Heated Broccoli Juice. J. Agric. Food Chem..

[43] Wu S.-C., Wang F.-J., Pan C.-L. (2010). The comparison of antioxidative properties of seaweed oligosaccharides fermented by two lactic acid bacteria. J. Mar. Sci. Technol..

[44] Pereira A.L.F., Maciel T.C., Rodrigues S. (2011). Probiotic beverage from cashew apple juice fermented with *Lacticaseibacillus casei*. Food Res. Int..

[45] Ricci A., Bernini V., Maoloni A., Cirlini M., Galaverna G., Neviani E., Lazzi C. (2019). Vegetable By-Product Lacto-Fermentation as a New Source of Antimicrobial Compounds. Microorganisms.

[46] Giulio B.D., Orlando P., Barba G., Coppola R., Rosa M.D., Sada A., Prisco P.P.D., Nazzaro F. (2005). Use of alginate and cryo-protective sugars to improve the viability of lactic acid bacteria after freezing and freeze-drying. World J. Microbiol. Biotechnol..

[47] Reddy K.B.P.K., Awasthi S.P., Madhu A.N., Prapulla S.G. (2009). Role of Cryoprotectants on the Viability and Functional Properties of Probiotic Lactic Acid Bacteria during Freeze Drying. Food Biotechnol..

[48] Varga L., Szigeti J., Kovács R., Földes T., Buti S. (2002). Influence of a *Spirulina platensis* Biomass on the Microflora of Fermented ABT Milks During Storage (R1). J. Dairy Sci..

[49] Beheshtipour H., Mortazavian A.M., Haratian P., Darani K.K. (2012). Effects of *Chlorella vulgaris* and *Arthrospira platensis* addition on viability of probiotic bacteria in yogurt and its biochemical properties. Eur. Food Res. Technol..

[50] De Marco Castro E., Shannon E., Abu-Ghannam N. (2019). Effect of Fermentation on Enhancing the Nutraceutical Properties of *Arthrospira platensis* (Spirulina). Fermentation.

[51] Dave R.I., Shah N.P. (1997). Viability of yoghurt and probiotic bacteria in yoghurts made from commercial starter cultures. Int. Dairy J..

[52] Bianchi F., Careri M., Mangia A., Musci M. (2007). Retention indices in the analysis of food aroma volatile compounds in temperature-programmed gas chromatography: Database creation and evaluation of precision and robustness. J. Sep. Sci..

[53] sherena.johnson@nist.gov Standard Reference Data. https://www.nist.gov/srd.

[54] Ricci A., Cirlini M., Levante A., Dall’Asta C., Galaverna G., Lazzi C. (2018). Volatile profile of elderberry juice: Effect of lactic acid fermentation using *L. plantarum*, *L. rhamnosus* and *L. casei* strains. Food Res. Int..

[55] Cirlini M., Dall’Asta C., Silvanini A., Beghè D., Fabbri A., Galaverna G., Ganino T. (2012). Volatile fingerprinting of chestnut flours from traditional Emilia Romagna (Italy) cultivars. Food Chem..

[56] Goodner K.L. (2008). Practical retention index models of OV-101, DB-1, DB-5, and DB-Wax for flavor and fragrance compounds. LWT Food Sci. Technol..

[57] Tanaka T., Yamaguchi T., Katsumata R., Kiuchi K. (2003). Comparison of volatile components in commercial Itohiki-Natto by solid phase microextraction and gas chromatography. Nippon Shokuhin Kagaku Kogaku Kaishi.

[58] Mena P., Cirlini M., Tassotti M., Herrlinger K., Dall’Asta C., Del Rio D. (2016). Phytochemical Profiling of Flavonoids, Phenolic Acids, Terpenoids, and Volatile Fraction of a Rosemary (*Rosmarinus officinalis L*.) Extract. Molecules.

[59] Yamamoto M., Baldermann S., Yoshikawa K., Fujita A., Mase N., Watanabe N. (2014). Determination of Volatile Compounds in Four Commercial Samples of Japanese Green Algae Using Solid Phase Microextraction Gas Chromatography Mass Spectrometry. Sci. World J..

[60] Ricci A., Cirlini M., Guido A., Liberatore C., Ganino T., Lazzi C., Chiancone B. (2019). From Byproduct to Resource: Fermented Apple Pomace as Beer Flavoring. Foods.

[61] Dall’Asta C., Cirlini M., Morini E., Galaverna G. (2011). Brand-dependent volatile fingerprinting of Italian wines from Valpolicella. J. Chromatogr. A.

[62] Ong P.K.C., Acree T.E. (1999). Similarities in the Aroma Chemistry of Gewu1 rztraminer Variety Wines and Lychee (*Litchi chinesis Sonn*.) Fruit. J. Agric. Food. Chem..

[63] Yanagimoto K., Ochi H., Lee K.-G., Shibamoto T. (2004). Antioxidative Activities of Fractions Obtained from Brewed Coffee. J. Agric. Food Chem..

[64] Babushok V.I., Linstrom P.J., Zenkevich I.G. (2011). Retention Indices for Frequently Reported Compounds of Plant Essential Oils. J. Phys. Chem. Ref. Data.

[65] Brunton N.P., Cronin D.A., Monahan F.J. (2002). Volatile components associated with freshly cooked and oxidized off-flavours in turkey breast meat. Flavour Fragr. J..

[66] Cirlini M., Mena P., Tassotti M., Herrlinger K., Nieman K., Dall’Asta C., Del Rio D. (2016). Phenolic and Volatile Composition of a Dry Spearmint (*Mentha spicata* L.) Extract. Molecules.

[67] Schneider H., Gelpi E., Bennett E.O., Oró J. (1970). Fatty acids of geochemical significance in microscopic algae. Phytochemistry.

[68] Stoyanova L.G., Ustyugova E.A., Netrusov A.I. (2012). Antibacterial metabolites of lactic acid bacteria: Their diversity and properties. Appl. Biochem. Microbiol..

[69] Höckelmann C., Jüttner F. (2005). Off-flavours in water: Hydroxyketones and β-ionone derivatives as new odour compounds of freshwater cyanobacteria. Flavour Fragr. J..

[70] Do Nascimento T.C., Nass P.P., Fernandes A.S., Vieira K.R., Wagner R., Jacob-Lopes E., Zepka L.Q. (2020). Exploratory data of the microalgae compounds for food purposes. Data Brief.

[71] Van Durme J., Goiris K., De Winne A., De Cooman L., Muylaert K. (2013). Evaluation of the Volatile Composition and Sensory Properties of Five Species of Microalgae. J. Agric. Food Chem..

[72] Isleten Hosoglu M. (2018). Aroma characterization of five microalgae species using solid-phase microextraction and gas chromatography–mass spectrometry/olfactometry. Food Chem..

[73] Achyuthan K., Harper J., Manginell R., Moorman M. (2017). Volatile Metabolites Emission by In Vivo Microalgae—An Overlooked Opportunity?. Metabolites.

[74] Amàrita F., Fernàndez-Esplà D., Requena T., Pelaez C. (2001). Conversion of methionine to methional by Lactococcus lactis. FEMS Microbiol. Lett..

[75] Amárita F., Martínez-Cuesta C.M., Taborda G., Soto-Yárritu P.L., Requena T., Peláez C. (2002). Formation of methional by *Lactococcus lactis* IFPL730 under cheese model conditions. Eur. Food Res. Technol..

[76] Helinck S., Le Bars D., Moreau D., Yvon M. (2004). Ability of Thermophilic Lactic Acid Bacteria To Produce Aroma Compounds from Amino Acids. Appl. Environ. Microbiol..

[77] De Melo Pereira G.V., da Silva Vale A., de Carvalho Neto D.P., Muynarsk E.S., Soccol V.T., Soccol C.R. (2020). Lactic acid bacteria: What coffee industry should know?. Curr. Opin. Food Sci..

[78] El-Gendy S.M., Abdel-Galil H., Shahin Y., Hegazi F.Z. (1983). Acetoin and Diacetyl Production by Homo- and Heterofermentative Lactic Acid Bacteria. J. Food Prot..

[79] Milovanović I., Mišan A., Simeunović J., Kovač D., Jambrec D., Mandić A. (2015). Determination of Volatile Organic Compounds in Selected Strains of Cyanobacteria. J. Chem..

[80] Li H., Liu L., Zhang S., Cui W., Lv J. (2012). Identification of Antifungal Compounds Produced by *Lacticaseibacillus casei* AST18. Curr. Microbiol..

