# Supplementary material for: Solid-State Fermentation of Arthrospira platensis to Implement New Food Products: Evaluation of Stabilization Treatments and Bacterial Growth on the Volatile Fraction
Source: Foods. 2020 Dec 30;10(1):67. doi: 10.3390/foods10010067 (PMC7823266; doi:10.3390/foods10010067)
Supplement: Supplementary file 1 [file foods-10-00067-s001.pdf]

**Table 1.** Concentration (μg/g) of compounds identified in UV treated (UV) and sterilized (ST) Arthrospira platensis fermented with L. casei 2240 (2240) and L. rhamnosus GG (GG) and in controls (UV treated and sterilized but not fermented Arthrospira platensis samples) after 48 hours.

| | Content | | | | | | Factors | | |
|---|---|---|---|---|---|---|---|---|---|
| **Compounds** | **Cntr ST** | **ST_2240** | **ST_GG** | **Cntr UV** | **UV_2240** | **UV_GG** | **F** | **S** | **F*S** |
| *Aldehydes* | | | | | | | | | |
| Isobutyraldehyde | 0.01086 ± 0.00077 a | n.d | n.d | n.d | 0.00991 ± 0.00286 a | n.d | 0.250 | 0.250 | 0.010 |
| 2-Methylbutanal | 0.02264 ± 0.00332 a | n.d | n.d | n.d | n.d | n.d | | | |
| Isovaleraldehyde | 0.02728 ± 0.00156 a | n.d | n.d | n.d | 0.01423 ± 0.00123 a | 0.01244 ± 0.00212 a | 0.000 | 0.000 | 0.000 |
| Hexanal | 0.04523 ± 0.00028 a | n.d | n.d | n.d | n.d | 0.05297 ± 0.00836 a | 0.468 | 0.468 | 0.016 |
| Methional | n.d | n.d | n.d | n.d | 0.02289 ± 0.00137 a | 0.02489 ± 0.00693 a | 0.000 | 0.000 | 0.000 |
| Benzaldehyde | 0.17073 ± 0.03177 a | n.d | n.d | n.d | 0.13162 ± 0.04463 a | 0.10459 ± 0.03979 a | 0.177 | 0.177 | 0.000 |
| 2,5-Dimethyl Benzaldehyde | 0.02227 ± 0.00508 a | n.d | n.d | n.d | n.d | n.d | 0.000 | 0.000 | 0.000 |
| *Sum* | 0,29901 ± 0,03909 a | n.d | n.d | n.d | 0,17866 ± 0,04418 b | 0,19489 ± 0,05415 b | 0.020 | 0.020 | 0.000 |
| *Ketones* | | | | | | | | | |
| Acetone | 0.01123 ± 0.00081 b | 0.02461 ± 0.00831 a | 0.02813 ± 0.00570 a | n.d | 0.01672 ± 0.00171 a | 0.02103 ± 0.00361 a | 0.000 | 0.004 | 0.470 |
| 2-Butanone | 0.01008 ± 0.00039 b | 0.02021 ± 0.04432 a,b | 0.02278 ± 0.00127a | n.d | 0.02219 ± 0.00416 a | 0.01859 ± 0.00415 a | 0.000 | 0.013 | 0.036 |
| Diacetyl | 0.00826 ± 0.00037 c | 0.04522 ± 0.00953 b | 0.01092 ± 0.00049 c | 0.00890 ± 0.00259 c | 0.07766 ± 0.00882 a | 0.03668 ± 0.01107 b | 0.019 | 0.250 | 0.270 |
| 6-Methyl-2-Heptanone | 0.01997 ± 0.00221 a | 0.02115 ± 0.00177 a | 0.01611 ± 0.00087 a | n.d | 0.01649 ± 0.00236 a | 0.01329 ± 0.00273 a | 0.002 | 0.000 | 0.001 |
| 3-Octanone | n.d | 0.00977 ± 0.00437 b | 0.01493 ± 0.00125 b | 0.00992 ± 0.00104 b,c | 0.01973 ± 0.00201 a | 0.00951 ± 0.00236 b | 0.013 | 0.056 | 0.205 |
| 2-Octanone | 0.01026 ± 0.00174 a | 0.00875 ± 0.00058 b | 0.00562 ± 0.00014 c | n.d | n.d | n.d | 0.048 | 0.000 | 0.048 |
| Acetoin | n.d | n.d | 0.08577 ± 0.00331 a | n.d | 0.09083 ± 0.00784 a | 0.13555 ± 0.04519 a | 0.006 | 0.053 | 0.053 |
| 2,2,6-Trimethylcyclohexanone | 0.06032 ± 0.01269 a | 0.05366 ± 0.00037 a | 0.04938 ± 0.00039 a | 0.00878 ± 0.00147 b,c | 0.02672 ± 0.00404 b | 0.01958 ± 0.00567 b | 0.450 | 0.000 | 0.009 |
| Sulcatone | 0.07005 ± 0.01557 a | 0.06322 ± 0.00502 a | 0.05586 ± 0.00220 a | n.d | 0.02003 ± 0.00166 b | 0.02032 ± 0.00488 b | 0.217 | 0.000 | 0.002 |
| *Sum* | 0,19017 ± 0,03299 a | 0,24658 ± 0,02572 a | 0,28949 ± 0,01182 a | 0,02759 ± 0,00511 b | 0,29037 ± 0,02579 a | 0,27455 ± 0,07501 a | 0.000 | 0.012 | 0.005 |
| *Esters* | | | | | | | | | |
| Ethyl Acetate | 0.01140 ± 0.00099 a | 0.01519 ± 0.00094 a | 0.01766 ± 0.00128 a | 0.01378 ± 0.00274 a | 0.02233 ± 0.00424 a | 0.01843 ± 0.00328 a | 0.011 | 0.119 | 0.680 |
| Ethyl Caprylate | n.d | n.d | n.d | 0.00840 ± 0.00068 a | n.d | n.d | | | |
| Ethyl Decanoate | n.d | n.d | n.d | 0.01150 ± 0.00237 a | 0.01017 ± 0.00214 a | n.d | 0.220 | 0.007 | 0.220 |
| Phenethyl Acetate | 0.01769 ± 0.00765 a | n.d | n.d | n.d | n.d | n.d | | | |
| *Sum* | 0,02909 ± 0,00864 a | 0,01519 ± 0,00094 b | 0,01766 ± 0,00128 b | 0,03368 ± 0,00105 a | 0,03251 ± 0,00220 a | 0,01843 ± 0,00328 b | 0.021 | 0.105 | 0.573 |
| *Terpenes/Norisoprenoids* | | | | | | | | | |
| Para-Xylene | 0.18493 ± 0.03116 a | 0.13716 ± 0.04784 a | 0.11965 ± 0.05426 a | n.d | 0.06621 ± 0.03628 b | n.d | 0.594 | 0.000 | 0.061 |
| Myrcene | n.d | n.d | n.d | n.d | n.d | 0.02083 ± 0.00970 a | 0.362 | 0.362 | 0.362 |

| | | | | | | | | | |
|---|---|---|---|---|---|---|---|---|---|
| α-Cyclocitral | 0.03281 ± 0.00801 a | n.d | 0.01957 ± 0.00194 b | n.d | n.d | n.d | 0.016 | 0.000 | 0.016 |
| β-Cyclocitral | 0.12264 ± 0.03553 a | 0.11031 ± 0.01463 a | 0.09302 ± 0.00787 a | 0.01258 ± 0.00178 b | 0.03159 ± 0.00529 b | 0.02949 ± 0.00622 b | 0.861 | 0.000 | 0.042 |
| Safranal | 0.02946 ± 0.00566 a | 0.01532 ± 0.00125 b | 0.01348 ± 0.00088 b | n.d | n.d | n.d | 0.000 | 0.000 | 0.000 |
| α-Ionene | n.d | n.d | 0.01743 ± 0.00140 a | n.d | n.d | n.d | 0.215 | 0.215 | 0.215 |
| α-Ionone | 0.01013 ± 0.00336 ac | 0.01473 ± 0.00073 a | 0.01429 ± 0.00184 a | n.d | n.d | n.d | 0.014 | 0.000 | 0.014 |
| β-Ionone | 0.05990 ± 0.01559 a | 0.05848 ± 0.00595 a | 0.06004 ± 0.00680 a | 0.03442 ± 0.00188 a | 0.06854 ± 0.01867 a | 0.06269 ± 0.02362 a | 0.114 | 0.304 | 0.101 |
| β-Ionone 5,6-Epoxide | 0.02621 ± 0.00492 a | 0.02515 ± 0.00285 a | 0.02666 ± 0.00081 a | n.d | n.d | n.d | 0.896 | 0.000 | 0.896 |
| *Sum* | 0,46607 ± 0,10424 a | 0,36116 ± 0,07324 a | 0,36415 ± 0,01973 a | 0,04700 ± 0,00367 c | 0,16634 ± 0,05402 b | 0,11300 ± 0,03795 b | 0.872 | 0.000 | 0.013 |
| *Alcohols* | | | | | | | | | |
| Ethanol | 0.01358 ± 0.00236 b | 0.02375 ± 0.00463 b | 0.06260 ± 0.00130 a,b | 0.06514 ± 0.01110 a,b | 0.09513 ± 0.01047 a | 0.08824 ± 0.02770 a,b | 0.030 | 0.001 | 0.893 |
| Isobutyl Alcohol | n.d | n.d | 0.00951 ± 0.00174 a | n.d | n.d | n.d | 0.221 | 0.221 | 0.221 |
| Isoamyl Alcohol | n.d | 0.02253 ± 0.00194 a | 0.02454 ± 0.00207 a | n.d | n.d | n.d | 0.000 | 0.000 | 0.000 |
| 1-Pentanol | 0.01052 ± 0.00087 a | 0.00907 ± 0.00012 a | 0.00988 ± 0.00123 a | n.d | n.d | n.d | 0.139 | 0.000 | 0.139 |
| 1-Hexanol | 0.03083 ± 0.00603 b | n.d | 0.05230 ± 0.00343 b | 0.04333 ± 0.00407 b | 0.08744 ± 0.00542 a | 0.06764 ± 0.01372 a | 0.233 | 0.021 | 0.126 |
| 1-Octen-3-Ol | 0.12615 ± 0.03004 a | 0.12107 ± 0.00564 a | 0.08971 ± 0.00728 a | 0.03629 ± 0.00480 b | 0.06884 ± 0.00639 a | 0.06283 ± 0.01333 a | 0.650 | 0.000 | 0.023 |
| Benzyl Alcohol | 0.01042 ± 0.00184 a | 0.01950 ± 0.00286 a | 0.01971 ± 0.00396 a | 0.01548 ± 0.00291 a | n.d | 0.04949 ± 0.02987 a | 0.523 | 0.722 | 0.998 |
| *Sum* | 0,19150 ± 0,03642 a | 0,19592 ± 0,01107 a | 0,26826 ± 0,00140 a | 0,16024 ± 0,02289 b | 0,25141 ± 0,01176 a | 0,26820 ± 0,08387 a | 0.031 | 0.951 | 0.315 |
| *Furans* | | | | | | | | | |
| 2-Methylfuran | 0.00770 ± 0.00032 c | 0.01372 ± 0.00090 b | 0.02194 ± 0.00116 a | n.d | n.d | n.d | 0.009 | 0.000 | 0.009 |
| 3-Methylfuran | n.d | 0.01148 ± 0.00242 a | n.d | n.d | n.d | n.d | 0.223 | 0.223 | 0.223 |
| 2-Butylfuran | 0.00711 ± 0.00041 a | 0.00599 ± 0.00102 a,b | 0.00482 ± 0.00044 b | n.d | n.d | n.d | 0.021 | 0.000 | 0.021 |
| 2-Pentylfuran | 0.09652 ± 0.03597 a | 0.06390 ± 0.02915 a | 0.06414 ± 0.00848 a | n.d | 0.02122 ± 0.00268 a | 0.01525 ± 0.00683 a | 0.455 | 0.000 | 0.020 |
| *Sum* | 0,11133 ± 0,03670 a | 0,09508 ± 0,03348 a | 0,09090 ± 0,00689 a | n.d | 0,02122 ± 0,00268 b | 0,01525 ± 0,00683 b | 0.996 | 0.000 | 0.091 |
| *Hydrocarbons* | | | | | | | | | |
| 1,2,4,4-Tetramethylcyclopentene | n.d | 0.00929 ± 0.00062 a | n.d | n.d | n.d | n.d | 0.215 | 0.215 | 0.215 |
| 2,2,4,6,6-Pentamethylheptane | 0.02296 ± 0.00690 a | 0.02007 ± 0.00515 a | 0.01964 ± 0.00168 a | 0.01729 ± 0.00582 a | 0.02289 ± 0.00682 a | 0.02597 ± 0.01047 a | 0.620 | 0.893 | 0.222 |
| Ethyl Benzene | n.d | n.d | n.d | n.d | 0.09524 ± 0.06073 a | n.d | 0.403 | 0.403 | 0.403 |
| Tridecane | n.d | n.d | n.d | 0.01100 ± 0.00145 a | n.d | n.d | 0.000 | 0.000 | 0.000 |
| Tetradecane | n.d | n.d | n.d | 0.01741 ± 0.00036 b | 0.04182 ± 0.00798 a | 0.03469 ± 0.00746 a | 0.011 | 0.000 | 0.011 |
| 2,6,10-Trimethyltridecane | n.d | n.d | n.d | n.d | n.d | 0.02482 ± 0.00399 a | 0.311 | 0.311 | 0.311 |
| Pentadecane | 0.15499 ± 0.07599 c | 0.24355 ± 0.08369 c | 0.22075 ± 0.05417 c | 0.66333 ± 0.00284 b | 1.49391 ± 0.27536 a | 1.24060 ± 0.23169 a | 0.007 | 0.000 | 0.021 |
| Hexadecane | 0.15713 ± 0.04745 b | 0.22729 ± 0.05538 b | 0.19335 ± 0.02903 b | 0.35436 ± 0.00582 b | 0.70480 ± 0.13366 a | 0.60046 ± 0.10782 a | 0.009 | 0.000 | 0.047 |
| Heptadecane | 1.13962 ± 0.37660 c | 2.53983 ± 0.66277 c | 2.43265 ± 0.33095 c | 4.15299 ± 0.09135 b | 7.65492 ± 1.95460 a | 7.00994 ± 1.23021 a | 0.006 | 0.000 | 0.190 |

| | | | | | | | | | |
|---|---|---|---|---|---|---|---|---|---|
| 6,9-Heptadecadiene | n.d | n.d | n.d | 0.07147 ± 0.00742 a | n.d | n.d | 0.000 | 0.000 | 0.000 |
| N-Acetyl-4(H)-Pyridine | 0.04755 ± 0.00854 a | 0.01928 ± 0.00342 b | 0.03432 ± 0.00153 a | n.d | n.d | n.d | 0.011 | 0.000 | 0.011 |
| *Sum* | 1,58257 ± 0,52818 c | 3,11297 ± 0,80943 c | 2,95009 ± 0,41776 c | 5,29663 ± 0,08348 b | 10,04030 ± 2,42966 a | 8,95606 ± 1,59015 a | 0.007 | 0.000 | 0.131 |
| *Pyrazines* | | | | | | | | | |
| 2-Methylpyrazine | n.d | n.d | 0.01654 ± 0.00083 a | n.d | n.d | n.d | 0.214 | 0.214 | 0.214 |
| 2,5-Dimethylpyrazine | 0.03803 ± 0.00694 a | 0.03379 ± 0.00435 a | 0.03696 ± 0.00272 a | n.d | n.d | n.d | 0.461 | 0.000 | 0.461 |
| 2-Methyl-5-Ethylpyrazine | n.d | n.d | 0.03285 ± 0.00635 a | n.d | n.d | n.d | 0.221 | 0.221 | 0.221 |
| 2-Ethyl-6-Methylpyrazine | 0.02741 ± 0.01126 a | 0.02143 ± 0.00012 a | n.d | n.d | n.d | n.d | 0.097 | 0.002 | 0.097 |
| Trimethyl Pyrazine | 0.02836 ± 0.00290 b | 0.05547 ± 0.00244 a | 0.05250 ± 0.00204 a | n.d | 0.00541 ± 0.00085 d | 0.01996 ± 0.00521 c | 0.000 | 0.000 | 0.116 |
| 2,3-Dimethyl-5-Ethylpyrazine | 0.01556 ± 0.00391 a | 0.01944 ± 0.00326 a | 0.01980 ± 0.00359 a | n.d | n.d | n.d | 0.114 | 0.000 | 0.114 |
| Tetramethyl Pyrazine | 0.01873 ± 0.00355 a | 0.01948 ± 0.00053 a | 0.02146 ± 0.00411 a | 0.00909 ± 0.00280 b | n.d | 0.01411 ± 0.00340 b | 0.969 | 0.009 | 0.610 |
| *Sum* | 0,12808 ± 0,02856 a | 0,14961 ± 0,00394 a | 0,18012 ± 0,01964 a | 0,00909 ± 0,00280 b | 0,00541 ± 0,00085 b | 0,03407 ± 0,00852 b | 0.060 | 0.000 | 0.270 |
| **Sulfur comp.** | | | | | | | | | |
| Dimethyl Disulfide | 0.05171 ± 0.02780 a | 0.03838 ± 0.02634 a | 0.01534 ± 0.00246 a | n.d | n.d | n.d | 0.171 | 0.001 | 0.171 |
| 2-Ethyl-4-Methylthiazole | n.d | n.d | n.d | n.d | n.d | 0.02147 ± 0.00262 a | 0.308 | 0.308 | 0.308 |
| Dimethyl Trisulfide | 0.01310 ± 0.00482 a | 0.01702 ± 0.01053 a | n.d | n.d | 0.03145 ± 0.01865 a | n.d | 0.575 | 0.765 | 0.315 |
| *Sum* | 0,06481 ± 0,03263 a | 0,05540 ± 0,03687 a | 0,01534 ± 0,00246 b | n.d | 0,03145 ± 0,01865 a | 0,02147 ± 0,00262 b | 0.912 | 0.019 | 0.060 |
| **Total aromatic compounds** | 3.063 ± 0.847 b | 4.232 ± 0.919 b | 4.176 ± 0.476 b | 5.574 ± 0.048 b | 11.018 ± 2.542 a | 9.896 ± 1.844 a | 0.008 | 0.001 | 0.670 |

Data are reported as average ± SD; letters indicate analogies and differences on the basis of one-way ANOVA, Tukey's test ($p \leq 0.05$); p-values obtained for two-way ANOVA, Tukey's test for the considered factors (F = fermentation, S = sterilization, F*S = interaction). ND compounds not detected.

**Table S2**. Concentration (μg/g) of odor type identified in UV treated (UV) and sterilized (ST) Arthrospira platensis fermented with L. casei 2240 (2240) and L. rhamnosus GG (GG) and in controls (UV treated and sterilized but not fermented Arthrospira platensis samples) after 48 hours

| | Content | | | | | | Factors | | |
|---|---|---|---|---|---|---|---|---|---|
| **Odor type** | **Cntr ST** | **ST 2240** | **ST GG** | **Cntr UV** | **UV 2240** | **UV GG** | **F** | **S** | **F*S** |
| Aldehydic/Ethereal | 0.104 ± 0.000 a,b | 0.105 ± 0.010 a,b | 0.157 ± 0.004 a | 0.079 ± 0.014 b | 0.197 ± 0.024 a | 0.172 ± 0.042 a | 0.00 | 0.44 | 0.05 |
| Sulfurous | 0.065 ± 0.033 a | 0.055 ± 0.037 a | 0.015 ± 0.002 a | n.d | 0.031 ± 0.019 a | n.d | 0.65 | 0.02 | 0.16 |
| Green/Herbal | 0.302 ± 0.056 a | 0.209 ± 0.011 a | 0.225 ± 0.004 a | 0.098 ± 0.011 b | 0.226 ± 0.019 a | 0.237 ± 0.046 a | 0.21 | 0.00 | 0.00 |
| Buttery/Waxy | 0.008 ± 0.000 c | 0.045 ± 0.010 b | 0.097 ± 0.003 a | 0.029 ± 0.001 b | 0.179 ± 0.018 a | 0.172 ± 0.056 a | 0.00 | 0.01 | 0.05 |
| Spicy | 0.007 ± 0.000 a | 0.006 ± 0.001 a | 0.005 ± 0.000 a | n.d | n.d | 0.021 ± 0.010 a | 0.44 | 0.85 | 0.29 |
| Fruity | 0.519 ± 0.132 a | 0.263 ± 0.052 b | 0.277 ± 0.023 b | 0.013 ± 0.002 c | 0.204 ± 0.054 b | 0.170 ± 0.053 b | 0.31 | 0.00 | 0.00 |
| Floral | 0.098 ± 0.028 a | 0.093 ± 0.010 a | 0.094 ± 0.005 a | 0.050 ± 0.005 a | 0.069 ± 0.019 a | 0.112 ± 0.053 a | 0.37 | 0.21 | 0.26 |
| Nutty/Roasted | 0.158 ± 0.032 a | 0.163 ± 0.005 a | 0.202 ± 0.018 a | 0.009 ± 0.003 b | 0.005 ± 0.001 b | 0.056 ± 0.011 b | 0.18 | 0.00 | 0.93 |
| Alkane | 1.452 ± 0.500 b | 3.011 ± 0.802 b | 2.847 ± 0.414 b | 5.199 ± 0.098 b | 9.895 ± 2.368 a | 8.886 ± 1.575 a | 0.01 | 0.00 | 0.13 |
| Fermented | 0.011 ± 0.001 b | 0.032 ± 0.002 a | 0.034 ± 0.001 a | n.d | n.d | n.d | 0.00 | 0.00 | 0.00 |
| NC (not classified) | 0.278 ± 0.052 a | 0.197 ± 0.058 a | 0.174 ± 0.003 a | 0.089 ± 0.013 a | 0.184 ± 0.096 a | 0.051 ± 0.014 a | 0.48 | 0.01 | 0.19 |

Data are reported as average ± SD; letters indicate analogies and differences on the basis of one-way ANOVA, Tukey's test ($p \leq 0.05$); p-values obtained for two-way ANOVA, Tukey's test for the considered factors (F = fermentation, S = sterilization, F*S = interaction). ND compounds not detected.
